# TYK2 Variants in B-Acute Lymphoblastic Leukaemia

**DOI:** 10.3390/genes11121434

**Published:** 2020-11-28

**Authors:** Edgar Turrubiartes-Martínez, Irene Bodega-Mayor, Pablo Delgado-Wicke, Francisca Molina-Jiménez, Diana Casique-Aguirre, Martín González-Andrade, Inmaculada Rapado, Mireia Camós, Cristina Díaz-de-Heredia, Eva Barragán, Manuel Ramírez-Orellana, Beatriz Aguado, Ángela Figuera, Joaquín Martínez-López, Elena Fernández-Ruiz

**Affiliations:** 1 Molecular Biology Unit, University Hospital La Princesa and Research Institute (IIS-IP), Diego de León 62, 28006 Madrid, Spain; edgar.turrubiartes@uaslp.mx (E.T.-M.); irenebodega@gmail.com (I.B.-M.); pablodelwik@gmail.com (P.D.-W.); frmolina@gmail.com (F.M.-J.); 2 School of Medicine, Latin University of Mexico (ULM), Avenida San Jose 100, 38085 Celaya, Mexico; diana.casique@gmail.com; 3 Biochemistry Department, Biosensors and Molecular Modelling Lab, Autonomous National University of México (UNAM), 04510 Mexico City, Mexico; martin@bq.unam.mx; 4 Haematology Department, University Hospital 12 Octubre and Research Institute (i+12), Avenida de Córdoba s/n., 28041 Madrid, Spain; inmaculada.rapado@salud.madrid.org (I.R.); jmarti01@med.ucm.es (J.M.-L.); 5 Haematology Department, University Hospital Sant Joan de Déu and Research Institute (IRSJD), Passeig Sant Joan de Déu 2, 08950 Esplugues de Llobregat, Spain; mcamos@hsjdbcn.org; 6 Paediatric Haematology and Oncology Department, University Hospital Vall d’Hebron and Research Institute (VHIR), Passeig de la Vall d’Hebron 119-129, 08035 Barcelona, Spain; crdiaz@vhebron.net; 7 Molecular Biology Lab, Clinical Analysis Department, University and Polytechnic Hospital and IIS La Fe, Avenida de Fernando Abril Martorell 106, 46026 Valencia, Spain; barragan_eva@gva.es; 8 Paediatric Haematology and Oncology Department, Paediatric University Hospital Niño Jesús (IIS-IP), Avenida de Menéndez Pelayo 65, 28009 Madrid, Spain; manuel.ramirez@salud.madrid.org; 9 Haematology Department, University Hospital La Princesa (IIS-IP), 28006 Madrid, Spain; baguadobueno@gmail.com (B.A.); a.figueraalvarez@gmail.com (Á.F.); 10 Spanish National Cancer Research Centre (CNIO), 28029 Madrid, Spain; 11 Faculty of Medicine, Complutense University of Madrid, 28040 Madrid, Spain; 12 Faculty of Medicine, Autonomous University of Madrid, 28049 Madrid, Spain

**Keywords:** B-cell precursor acute lymphoblastic leukaemia, TYK2 variants, IFNα, IFNα/β receptor alpha chain (IFNAR1), next-generation sequencing, molecular dynamics, TYK2 expression, immune surveillance

## Abstract

B-cell precursor acute lymphoblastic leukaemia (B-ALL) is a malignancy of lymphoid progenitor cells with altered genes including the Janus kinase (JAK) gene family. Among them, tyrosine kinase 2 (TYK2) is involved in signal transduction of cytokines such as interferon (IFN) α/β through IFN−α/β receptor alpha chain (IFNAR1). To search for disease-associated *TYK2* variants, bone marrow samples from 62 B-ALL patients at diagnosis were analysed by next-generation sequencing. *TYK2* variants were found in 16 patients (25.8%): one patient had a novel mutation at the four-point-one, ezrin, radixin, moesin (FERM) domain (S431G) and two patients had the rare variants rs150601734 or rs55882956 (R425H or R832W). To functionally characterise them, they were generated by direct mutagenesis, cloned in expression vectors, and transfected in TYK2-deficient cells. Under high-IFNα doses, the three variants were competent to phosphorylate STAT1/2. While R425H and R832W induced STAT1/2-target genes measured by qPCR, S431G behaved as the kinase-dead form of the protein. None of these variants phosphorylated STAT3 in in vitro kinase assays. Molecular dynamics simulation showed that TYK2/IFNAR1 interaction is not affected by these variants. Finally, qPCR analysis revealed diminished expression of *TYK2* in B-ALL patients at diagnosis compared to that in healthy donors, further stressing the tumour immune surveillance role of TYK2.

## 1. Introduction

B-cell precursor acute lymphoblastic leukaemia (B-ALL) is a neoplasia of lymphoid progenitor cells, characterised by large biological and clinical heterogeneity. Despite recent advances in genomic studies, there is still a limited understanding of the genetic basis of the disease [1,2]. B-ALL is the most frequent childhood cancer and accounts for 25% of adult acute leukaemia, remaining an important cause of morbidity [3]. Mutations in the non-receptor Janus kinase (JAK) 2 are important prognostic biomarkers in B-ALL [4,5], but the role of mutations in other members of this tyrosine kinase gene family is not well characterised.

JAK proteins have several domains that share seven homology regions (JH1–JH7) [6]. The carboxyl-terminal domain contains the active kinase domain (JH1) and the pseudokinase domain (JH2) that regulates catalytic activity. The N-terminal half of JAK proteins is most divergent and contains an src-homology 2 (SH2) domain (JH3 and part of JH4) with unknown function and a four-point-one, ezrin, radixin, moesin (FERM) homology domain (part of JH4 and JH5-JH7). The SH2 and FERM domains mediate cytokine receptor binding and regulate kinase activity. Tyrosine kinase 2 (TYK2) is a member of the JAK family involved in cytokine signal transduction in immune and haematopoietic cells [7,8] and is able to associate to five different low-affinity cytokine receptor subunits, i.e., the interferon (IFN) α/β receptor alpha chain (IFNAR1), the interleukin (IL)-12Rβ1, the IL-10Rβ, the IL-6Rα, and the IL-13Rα, and collaborates with JAK1 or JAK2 transducing cytokine signals [9]. Activation of the JAK–cytokine receptor complex leads to the recruitment and JAK-mediated phosphorylation of the signal transducers and activators of transcription (STAT) family of transcription factors, whose subsequent dimerisation and nuclear translocation induces target gene expression [10].

TYK2 deficiency in humans is associated with increased susceptibility to bacterial and viral infection [11,12]. Altered IL-6 responses, hyper IgE syndrome and allergic asthma have also been described [13]. Loss of TYK2 in murine models results in reduced cytokine responses [14,15]. Moreover, *Tyk2*-null mice have increased predisposition to B lymphoid tumour formation due to an impaired immune surveillance [16]. Over the last years, several reports on the relationship between TYK2 and cancer have emerged, although its role is not clearly established [17,18]. In humans, fusion proteins containing TYK2 have been identified in patients with haematopoietic malignancies [18] and germline TYK2-activating mutations have been described in two cases of secondary B-ALL [19]. A *TYK2* single-nucleotide polymorphism (SNP) in the kinase domain (P1104A) has been associated with different types of tumours [20] and has been found in about 7% of acute myeloid leukaemia (AML) patients [21]. TYK2 can also mediate drug resistance to the JAK2 inhibitor, ruxolitinib, via the formation of drug-resistant TYK2/JAK2 heterodimers [22]. Given the importance of a fully functional immune response for appropriate tumour surveillance, and that those immune responses rely on JAK-dependent signal transduction, it is of high relevance to elucidate the role of the interaction of cytokine receptors, JAK proteins, and their target genes. In this context, the search and functional characterisation of TYK2 variants could clarify their relevance in disease development and/or progression.

In this study, we analysed bone marrow samples from 62 B-ALL patients at diagnosis by next-generation sequencing (NGS), searching for disease-associated TYK2 variants. We found a novel variant, TYK2 S431G, together with the rare polymorphisms TYK2 R425H (rs150601734) and TYK2 R832W (rs144995884). The present study describes a functional characterisation of these variants, finding differences in their kinase activity and IFNα-mediated signalling. Additionally, we found a diminished *TYK2* expression in B-ALL patients compared to healthy donors. Taking together these results, we propose a role of TYK2 in the pathogenesis of B-ALL through the alteration of IFNα signalling.

## 2. Material and Methods

### 2.1. Patients and Data Collection

A total of 88 B-ALL patients referred from six Spanish centres to the Molecular Biology Unit at La Princesa University Hospital, Spain, between January 2013 and December 2015 were eligible for this study. Sixty-seven patients were children (76.14%, <18 years) and 21 were adults (23.86%, >18 years). The diagnosis of B-ALL was based on morphological, immunophenotypic, and genetic features of leukaemic blast cells, as described previously [23].

### 2.2. Ethics Statement

Written informed consent was obtained from each patient or from legal guardians before patients entered the study in all hospitals involved. The study was approved by the Clinical Research Ethics Committee of La Princesa University Hospital (approval ID: PI-655) on 7 November 2012.

### 2.3. DNA Isolation and Next-Generation Sequencing Assay (NGS)

Amplicon-based NGS was performed on 62 samples obtained from untreated patients at diagnosis (54 patients were children (87.10%, <18 years) and eight were adults (12.90%, >18 years) and five B-cell lines (Granta-519 and Jeko1: leukaemic transformation of mantle cell lymphoma (MCL); Ramos: Burkitt’s lymphoma; Mino: MCL and REH: B-ALL) from ATCC. Genomic DNA was extracted from bone marrow samples with the MagNA Pure LC DNA Isolation Kit (Roche Diagnostics, Basel, Switzerland) following the manufacturer’s instructions. An amplicon-based NGS assay was performed with 45 amplicons ranging from 275 to 144 bp (AmpliSeq; ThermoFisher Scientific, Waltham, MA, USA) covering the exon and flanking intron regions of *TYK2* (RefSeq: NM_003331.4) with an overall coverage of 93.98%, using the Ion Torrent Sequencing Platform (Applied Biosystems, Cheshire, UK) (Table S1). Data were validated by Sanger using TaqMan^®^ SNP Genotyping Assays (Applied Biosystems) or PGM Sequencing Platform (Applied Biosystem, Cheshire, UK). Amplicon library pooling, purification, emulsion PCR, sequencing, validation of variants, data processing, and analysis was performed as previously described [5]. The novel variant S431G was annotated in GeneBank (BankIt2289375 ENST00000525621.6 MN842724).

### 2.4. Site-Direct Mutagenesis, Plasmids, Cell Culture, and Transfection

TYK2 variants were obtained by oligonucleotide-mediated site-direct mutagenesis (ThermoFisher Scientific) of wild-type *TYK2* cloned in the pRc-CMV–neo eukaryotic expression vector (pRc-Tyk2) provided by Dr. Pellegrini (Institut Pasteur, Paris, France) [7] and the final constructs were sequenced. The control variants pRc-TYK2 K930R (kinase dead) and pRc-TYK2 V678F (catalytically hyperactive) were also provided by Dr. Pellegrini, together with the 293T and TYK2-deficient, IFNα-unresponsive human fibrosarcoma 11,1 cells (also called U1A) [7]. The recessive mutant 11,1 (U1A) was obtained from the parental 2fTGH cell line, derived by genetic approach to isolate regulatory mutations in the IFNα signalling pathway [24]. Mycoplasma free 293T and U1A cells were grown in Dulbecco’s modified Eagle medium (DMEM) (Gibco; ThermoFisher Scientific) containing 10% heat-inactivated foetal calf serum (FCS). Transfection of pRc-TYK2 mutants was performed with FuGENE6 (Roche Applied Science, Penzberg, Germany) following the manufacturer´s instructions. Final, stable TYK2-expressing clones were selected in 400 μg/mL of Geneticin G418, and resistant colonies were ring-cloned two weeks later and expanded.

### 2.5. Western Blot and Immunoprecipitation Analysis

Cells were starved for 2 h in serum-free DMEM before IFNα–2b stimulation for 15 min with concentrations ranging from 10 to 500 pM (Immunotools GmbH; Friesoythe, Germany). Cells were lysed in RIPA buffer using a protease and phosphatase inhibitor cocktail (ThermoFisher Scientific). Protein content was determined by a BCA Protein Assay Kit (ThermoFisher Scientific), and 30 μg was separated by 7% SDS-PAGE, analysed by Western blot and revealed using ECL detection reagents (ThermoFisher Scientific). For re-probing, blots were stripped with Restore plus Western blot stripping buffer (Cultek, Madrid, Spain) for 10 min at room temperature. The following Abs were used: anti phospho (p)-STAT1 (Y701), anti pSTAT2 (Y690), anti p-STAT3 (Y705), anti p-TYK2 (YY1054/55), directed to the activation loop tyrosine (Cell Signalling Technology, Beverly, CA, USA), anti TYK2 mAb T10-2 (Hybridolab, Institut Pasteur, kindly gifted by Dr. S. Pellegrini) raised against a GST fusion protein containing amino acids 289–451 of human TYK2, anti STAT3 (Santa Cruz Biotechnology Inc., Santa Cruz, CA, USA) and anti Tubulin (Sigma-Aldrich, St. Louis, MO, USA).

### 2.6. In Vitro Kinase Assay

An in vitro kinase assay was performed as described [25]. Briefly, cells were lysed in 50 mM Tris (pH 6.8), 0.5% Nonidet P-40, 200 mM NaCl, 10% glycerol, and 1 mM EDTA in presence of protease inhibitors. TYK2 was immunoprecipitated from 2 mg lysate using non-commercial R5-9 polyclonal Ab (a gift from S. Pellegrini, Institut Pasteur, Paris, France) and 40 μL protein A/G PLUS-Agarose (Santa Cruz Biotechnology, Inc.) with slow rotation at 4 °C overnight. Immunocomplexes were washed three times in buffer 1 (50 mM Tris (pH 6.8), 400 mM NaCl, 0.5% Triton X-100, and 1 mM EDTA), once in buffer 2 (50 mM Tris (pH 6.8) and 200 mM NaCl), and once in kinase buffer (50 mM HEPES (pH 7.6) and 10 mM MgCl_2_). The kinase reaction was carried out in kinase buffer, in the presence of 0.06 μg of recombinant (r) STAT3 (SignalChem Lifesciences Corporation, British Columbia, Canada), and with or without 30 μM ATP at 30 °C for 5 min in a total volume of 30 μL. The reaction was finished by boiling in Laemmli buffer. Half of the sample was loaded for SDS-PAGE and transferred to a nitrocellulose membrane, and phosphorylated products were analysed by Western blotting.

### 2.7. Expression Analysis

For *TYK2* expression analysis, 88 B-ALL patients were compared to 105 healthy donors. Reverse-transcriptase polymerase chain reaction (RT-PCR) was performed using Superscript R Vilo TM cDNA kit (Applied Biosystems) with 1 μg total RNA isolated from peripheral blood mononuclear cells (PBMCs), isolated by Ficoll from 10 mL of peripheral blood, in the case of healthy donors, or bone marrow at diagnosis, in the case of patients, using Ultraspec (Biotecx, Houston, TX, USA). Complementary DNA (cDNA) samples were diluted 1:10 before amplification by qPCR using TaqMan Fast-Advanced MMix (Applied Biosystems) in a CFX384 Real-Time System (Roche Diagnostics). The following TaqMan assays were used: Hs00177464_m1 (*TYK2*), Hs03929097_g1 (*GAPDH*) (ThermoFisher Scientific) following the manufacturer’s protocols. For each sample, relative mRNA levels were determined using the comparative Ct (ΔΔCt) method (normalised to *GAPDH*). In the case of U1A transfected cells, different clones were incubated 1 and 3 h with 500 pM of IFN after 2 h of serum starvation, and total RNA isolation and RT-PCR were performed as detailed above. TaqMan assays used were Hs00971965_m1 for interferon regulatory factor 1 (*IRF1*), Hs00973635_m1 for 2’-5’-oligoadenylate synthetase 1 (*OAS1*), Hs02330328_s1 for suppressor of cytokine signalling 3 (*SOCS3*), and Hs01065498_m1 for serine/threonine-protein kinase Pim-1 (*PIM1*) (ThermoFisher Scientific).

### 2.8. Structural Analysis of R425H and S431G TYK2 Variants Interacting with IFNAR1 and Molecular Dynamics Analysis

The crystallographic data of human TYK2 and the structural complexes were obtained from the Protein Data Bank (PDB ID: 4PO6). Visualization, structural analysis of the protein and the “in silico” mutant were made with PyMOL [26]. The TYK2 wild type (WT)–IFNAR1, TYK2 R245H–IFNAR1, and TYK2 S431G–IFNAR1 complexes were verified, cleaned, and ordered with the pdb4amber scrip before starting the preparation in order to generate suitable topologies from the LEaP module of AMBER 19 [27,28]. Each structure and complex was subjected to the following protocol: hydrogen’s and other missing atoms were added using the LEaP module with the leaprc.protein.ff19SB parameter set, Cl^−^ or K^+^ counterions were added to neutralise the system, the complexes were then solvated in an octahedral box of explicit TIP3P model water molecules localising the box limits at 12 Å from the protein surface. All calculations were made using graphics processing unit (GPU)-accelerated molecular dynamic (MD) engine in AMBER (pmemd.cuda) [29]. The protocol consisted in performing a minimisation of the initial structure, followed by 50 ps heating and pressure equilibration at 315 K and 1.0 atm pressure, respectively. Finally, the system was equilibrated with 500 ps before starting the production of MD, which consisted of 50 ns for each complex. Frames were saved at 10 ps intervals for subsequent analysis. All analyses were done using CPPTRAJ [30] and binding free energies calculated by molecular mechanics/Poisson–Boltzmann surface area (MM/PBSA) [31]. The calculations of root mean square deviation (RMSD) and root mean square fluctuation (RMSF) were made, considering C, CA, and N atoms.

### 2.9. Statistical Analysis

When analysing STAT target genes’ expression, cell line intrinsic differences between time conditions were analysed by two-way ANOVA, performing post hoc Bonferroni tests for intergroup differences (* vs. 0 h from each *TYK2* variant; # vs. wild type (WT)’s same time condition. For * and #: * *p* < 0.05; ** *p* < 0.01; *** *p* < 0.001; **** *p* < 0.0001). Differences in *TYK2* expression between healthy controls and B-ALL patients were evaluated using the Mann–Whitney U test. Association between *TYK2* expression and patient age was estimated using Spearman correlation (ρ). Prior to the correlation analysis, the Shapiro–Wilk test was performed. Overall survival (OS) curves were estimated by the Kaplan–Meier method, and the log-rank test or Tarone–Ware test. Overall survival risk by group was estimated using Cox regression. The χ^2^ and Fisher’s exact two-sided tests were used for comparisons between categorical variables, and the Wilcoxon rank sum test or t test was used for continuous variables. *p*-values < 0.05 were considered significant. The statistical analysis was carried out with SPSS program (Statistical Package for Social Sciences Inc., Chicago, IL, USA; version 22.0) and the version 6.05 of the program GraphPad Prism (GraphPad Software Inc, San Diego, CA, USA), also used for graphical representation.

## 3. Results

### 3.1. *TYK2* Non-Synonymous Variants Found in B-ALL Patients

We performed ultra-deep sequencing of bone marrow samples at diagnosis from 62 B-ALL patients and five B-cell lines (Granta-519, Jeko1, Ramos, Mino, and REH). The blast count in the patients ranged from 50% to 96% with a median of 90%. We found *TYK2* non-synonymous variants in 16 patients (25.8%) (Table S2). One of them had a non-described substitution (Table 1). *TYK2* variants were not associated with survival or any main clinical characteristic of patients, including genetic abnormalities (data not shown). None of the *TYK2* variants were found in the five B-cell lines analysed, whereas seven were detected in B-ALL samples: located in exon 9 (JH4), rs150601734 that causes arginine-to-histidine substitution (p.Arg425His) (TYK2 R425H) in one patient; in exon 15 (JH2), rs12720356 (p.Ile684Ser) (TYK2 I684S) in eleven patients (17.74%), and rs55882956 (p.Arg703Trp) (TYK2 R703W) in one case; in exon 18 (JH2), rs144995884 (p.Arg832Trp) (TYK2 R832W) was detected in one patient.

Two patients bearing rs12720356 (p.Ile684Ser) (TYK2 I684S) also had rs34536443 (p.Pro1104Ala) (TYK2 P1104A), located in exon 23 (JH1). Finally, one patient showed rs55886939 (p.Glu1163Gly) (TYK2 E1163G) in exon 25. In addition to these SNPs, we found a novel variant located in exon 9, which results in the replacement of serine by glycine (p.S431G) (TYK2 S431G) in one patient. This variant is localised in the FERM domain (JH4) of the protein and has not been previously described.

The variant allele frequency (VAF) obtained for the total variants analysed ranged from 32% to 54% with a median of 49% (Table S2).

### 3.2. TYK2 Variants Present Differences in IFN Signalling Response and in STAT-Target Genes Induction

**Figure 1:**
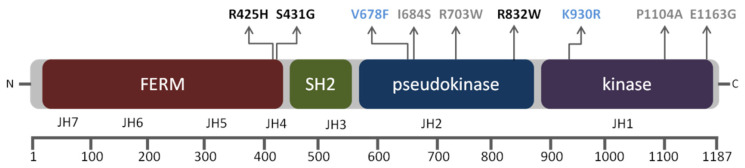
Schematic representation of structural and functional domains of tyrosine kinase 2 (TYK2) protein with the non-synonymous variants detected in this study (functional characterised variants in black; artificial mutant controls in blue: kinase-dead form of the protein (TYK2 K930R) and the catalytically hyperactive form (TYK2 V678F). Boxes illustrate functional domains from N- to C-terminus and the corresponding Janus kinase (JAK) structural homology domains (JH1–JH7) are marked below. Amino acid numbers are shown in the lower bar scale according to the human TYK2 protein sequence (RefSeq: NM_003331.4; FERM domain: 26–431, SH2 domain: 452–551, pseudokinase domain: 551–875, and kinase domain: 897–1176).

We decided to evaluate the effect in protein function of the novel variant TYK2 S431G, the very rare TYK2 R425H and TYK2 R832W that were not characterised previously (Figure 1).

**Figure 2:**
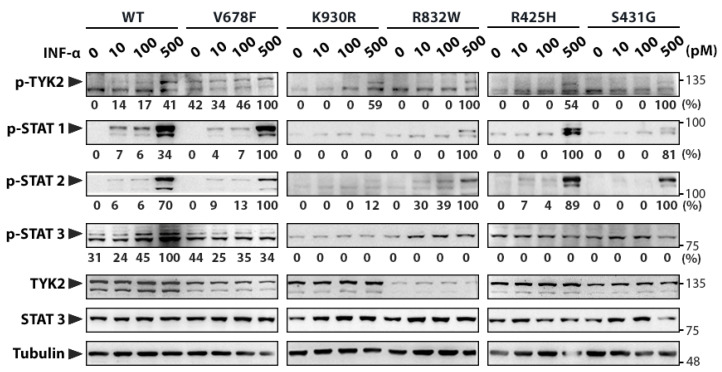
Functional analyses of TYK2 variants. Human TYK2-deficient U1A cell line was transfected with the different TYK2 variants and clones stably expressing these variants were selected. Clones were stimulated with the indicated doses of IFNα for 15 min, and the level of phosphorylation of TYK2 variants and STAT1–3 transcription factors was assessed by Western blot. Arrowheads point out the size of specific proteins and molecular weight markers (kDa) are noted on the right. Quantification of phosphorylated proteins is relative to TYK2 total protein (to account for TYK2 expression differences among cell lines) and then to Tubulin (shown under each lane/membrane). Values are expressed as percentage of the highest value of each membrane. WT: wild-type TYK2; V678F: catalytically hyperactive TYK2; K930R: kinase-dead TYK2 (ATP-binding site mutant); R832W, R425H, and S431G: disease-associated TYK2 variants. A representative result of three independent experiments is shown (Figure S2).

For this purpose wild-type (WT) *TYK2* and the three engineered *TYK2* mutant cDNAs were cloned in the pRc/CMV-neo expression vector, as well as the kinase-dead form of the protein (TYK2 K930R) as a negative control, and the catalytically hyperactive form (TYK2 V678F) as a positive control [39]. First, we decided to compare the basal auto-phosphorylation activity of TYK2 variants by expressing them in 293T cells (Figure S1A). Cell lysates were analysed by Western blot using the phospho-specific antibody directed to the activation-loop tyrosines (Tyr 1054–1055). TYK2 WT and the catalytically hyperactive TYK2 V678F were phosphorylated on tyrosine and activated STAT3. However, neither the catalytically inactive form of the protein (TYK2 K930R) nor the three variants (TYK2 R425H, TYK2 S431G, and TYK2 R832W) were auto-phosphorylated or able to activate STAT3. Then, the TYK2 variants were stably transfected in human TYK2-deficient U1A cells to test their phosphorylation state and signal capacity in response to IFNα. Cell clones were selected based on their TYK2 expression evaluated by Western blot (Figure S1B). The expression levels of the variants except K930R, were lower than those in WT. These cell clones were exposed for 15 min to increasing concentrations of IFNα to compare their phosphorylation state by Western blot (Figure 2). As expected, TYK2, STAT1, STAT2, and STAT3 were phosphorylated in response to IFNα in TYK2 WT and TYK2 V678F-transfected cells, whereas they remained non-phosphorylated in cells transfected with TYK2 K930R. TYK2 R425H and TYK2 R832W were able to mediate STAT2 phosphorylation in response to increasing doses of IFNα, but STAT1 became phosphorylated only at the highest dose of the cytokine (500 pM). On the other hand, TYK2 S431G only presented STAT1 and STAT2 phosphorylation at 500 pM of IFNα. Therefore, we concluded that these changes in the FERM and JH2 domains of TYK2 impaired both its ability to phosphorylate STAT3 and the activation of STAT1 in response to low and moderate doses of the cytokine.

**Figure 3:**
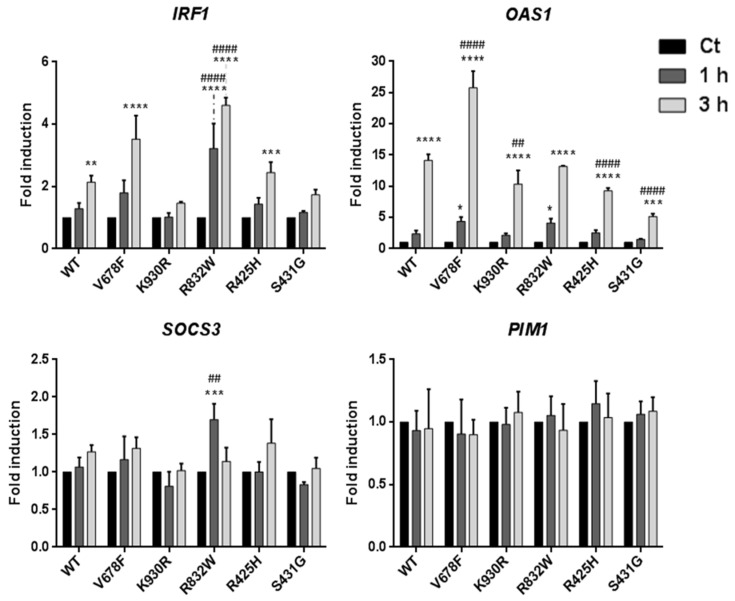
STAT-target gene expression induced by IFNα in U1A cells expressing TYK2 variants. Cells were treated with 500 pM IFNα. Total RNA was extracted, reverse-transcribed, and analysed by RT-qPCR for the indicated genes. Values were normalised to *GAPDH* and are expressed as *n*-fold versus untreated cells for each variant. Mean values of three independent experiments ± SD are shown. Two-way ANOVA was used for statistical analysis, performing post hoc Bonferroni tests for intergroup differences. * For each variant, comparison between the indicated time and untreated cells; # for each time condition, comparison between each variant and WT. For * and #: * *p* < 0.05; ** *p* < 0.01; *** *p* < 0.001; **** *p* < 0.0001.

To gain further insight into STAT biological activity in U1A cell clones under IFNα stimulation, we assessed the induction of IFNα-stimulated early genes after treating them for different times with 500 pM IFNα. Transcripts of *IRF1*, a STAT1-dependent gene; *OAS1*, a STAT1- and STAT2-dependent gene; and *SOCS3* and *PIM1*, both STAT3- and STAT5-dependent genes [39,40,41,42] were quantified by qPCR (Figure 3). TYK2 V678F cells treated with IFNα for 3h showed 4- and 27-fold induction of *IRF1* and *OAS1* transcripts, respectively, compared to untreated cells; whereas TYK2 WT showed half induction of both genes compared with the catalytically hyperactive variant. Surprisingly, considering *IRF1* induction, the TYK2 R832W expressing clone reached earlier a similar level to that showed by TYK2 V678F, whereas TYK2 R425H was comparable to TYK2 WT. In the case of *OAS1*, TYK2 R832W induced the gene in a similar way to the WT at 3 h, but the rest of the variants showed a significantly diminished induction after the same time of IFNα stimulation. In addition, significant induction was also observed after 1 h by both TYK2 V678F and TYK2 R832W. On the other hand, TYK2 S431G behaved as the kinase-dead TYK2 K930R for both genes. *SOCS3* and *PIM1* were not consistently induced at the times selected by any of the TYK2 variants with the exception of R832W, which reached a significant induction of *SOCS3* after 1 h of IFNα stimulation. This result further supports the finding that TYK2 R832W confers higher susceptibility to IFNα, whereas TYK2 S431G cells remain unable to appropriately respond to this cytokine.

### 3.3. In Vitro Kinase Activity of TYK2 Variants Is Impaired

**Figure 4:**
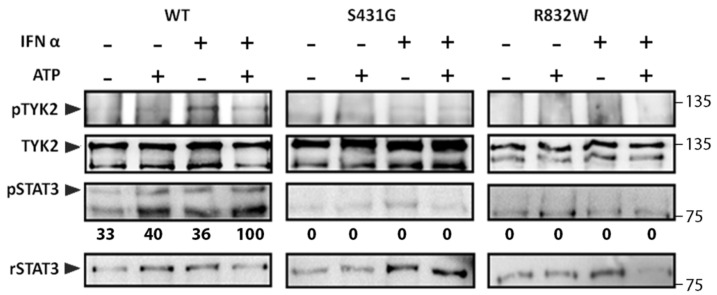
In vitro kinase activity of TYK2 WT, TYK2 S431G, and TYK2 R832W proteins. TYK2 was immunoprecipitated with anti-TYK2 Ab from non-stimulated cells or cells stimulated with IFNα (500 pM) for 15 min and subjected to in vitro kinase reaction in the presence or absence of 30 μM ATP for 5 min at 30 °C. Recombinant STAT3 (rSTAT3) was added to the reaction as exogenous substrate. The phosphorylation level of the indicated proteins was analysed by immunoblotting. STAT3 phosphorylation was represented relative to rSTAT3 and then to TYK2, to account for differences in the amount of immunoprecipitated TYK2 in each sample. Values are expressed as percentage of the highest value of each membrane. A representative result of three independent experiments is shown.

To further confirm STAT response when stimulating these TYK2 variants, we first compared the auto-phosphorylation ability of one of the FERM domain variants (TYK2 S431G) and the TYK2 R832W, localised at the pseudokinase domain, with that of TYK2 WT. To this end, the protein was immunoprecipitated with anti-TYK2 T10-2 mAb from cell lysates of each U1A cell clone and subjected to an in vitro kinase assay in the presence or absence of added ATP (Figure 4). Recombinant STAT3 (rSTAT3) was added to the reaction as exogenous substrate. The reaction product was analysed by immunoblotting with anti p-TYK2 and anti p-STAT3 Abs. In intact cells in the absence of ATP, no phosphorylation was detected in any of the assays, but when ATP was added to the reaction, p-TYK2 and p-STAT3 were detected in TYK2 WT reaction, whereas no signal was detected in the assays with TYK2 S431G or in the TYK2 R832W. When U1A cell clones were previously stimulated with IFNα, an increase in p-TYK2 was observed in TYK2 WT but not in TYK2 S431G or TYK2 R832W (Figure 4, right panel). Taken together, these data indicate that, in vitro, TYK2 S431G and TYK2 R832W have impaired basal and IFNα-induced auto-phosphorylation activity and are also unable to activate STAT3. Therefore, replacements of Ser431 by Gly in the FERM domain and Arg832 by Trp in the JH2 domain suppress TYK2 kinase activity.

### 3.4. Molecular Dynamics of the TYK2 R425H– and S431G–IFNAR1 Complex Interaction

**Figure 5:**
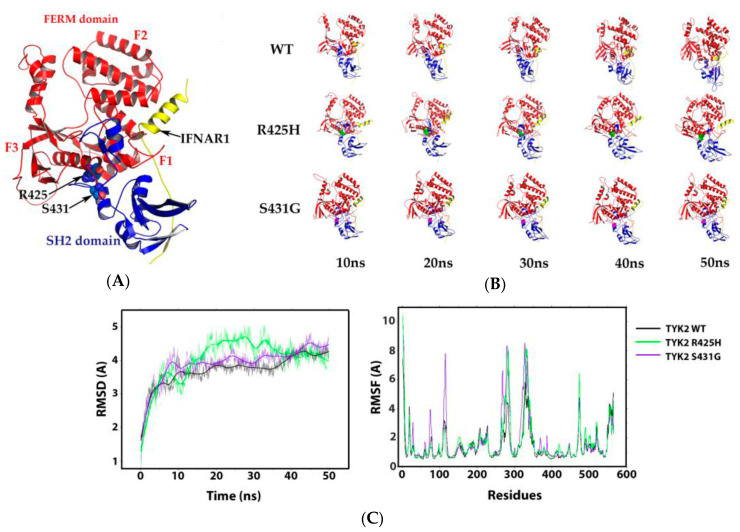
Interaction of four-point-one, ezrin, radixin, moesin (FERM) and src-homology 2 (SH2) domains of TYK2 with IFNAR1−α/β receptor alpha chain (IFNAR1). (**A**) 3D representation of the interaction of TYK2 FERM and SH2 domains with IFNAR1 (ID PDB: 4PO6). FERM domain is represented by subdomains (F1, F2, and F3) in red, SH2 domain in blue, and IFNAR1 in yellow; the sites of TYK2 R425 and S431 are represented with spheres in the protein structure and are indicated with black arrows. (**B**) Structural models from 0 to 50 ns of molecular dynamics (MD) of the TYK2–IFNAR1 complex, using the WT, R425H and S431G mutants. (**C**) Root mean square deviation (RMSD) and root mean square fluctuation (RMSF) of the TYK2 WT–IFNAR1, the TYK2 R425H–IFNAR1, and TYK2 S431G–IFNAR1 complexes. RMSD as a function of time (**left**) and RMSF vs. residues (**right**).

The FERM domain is involved in the scaffold formed by TYK2 together with IFNAR1, leading to an appropriate downstream signalling of the receptor complex [43]. However, according to the crystallographic structure of TYK2 interacting with IFNAR1 [44], this receptor mostly binds to the SH2 domain and the F1 and F2 subdomains of FERM (Figure 5A). The region where R425H and S431G mutations are located in the F3 subdomain of FERM domain is very distant from the binding site of IFNAR1, thus decreasing the likelihood that those point-mutations may affect the interaction of TYK2 with IFNAR. To confirm this point, the impact of S431G and R425H mutants on this protein interaction was determined by molecular dynamics (MD) analysis of the TYK2–IFNAR1 complex using the WT, the R425H and the S431G mutants (Figure 5B,C). Regarding the R832W mutant, it was not included in the MD studies, since it is found in the pseudokinase domain at 82.2 Å from the protein–peptide interaction under study (Figure S3).

For that purpose, the energy of the system was monitored as a function of time (Figure S4) establishing that from 10 ns it remains stable. The 50 ns structural models of MD of the complex showed that IFNAR1 remains bound to TYK2 WT, TYK2 R425H, and TYK2 S431G. The analysis of the MD trajectory (Figure 5C) showed a small difference in the RMSD between the WT and the two mutants, attributed mainly to the dynamics itself; likewise, the RMSF of both complexes overlap and discrepancies only appear in small regions (residues 286–311 and 346–365). The theoretical energy calculations are detailed (Table S3) and they gave a binding affinity (∆G_bind_) of −117.85, −122.81, and −126.49 for the complexes TYK2 WT–IFNAR1, TYK2 R425H–IFNAR1, and TYK2 S431G–IFNAR1, respectively.

### 3.5. *TYK2* Expression Analysis in B-ALL Patients

**Figure 6:**
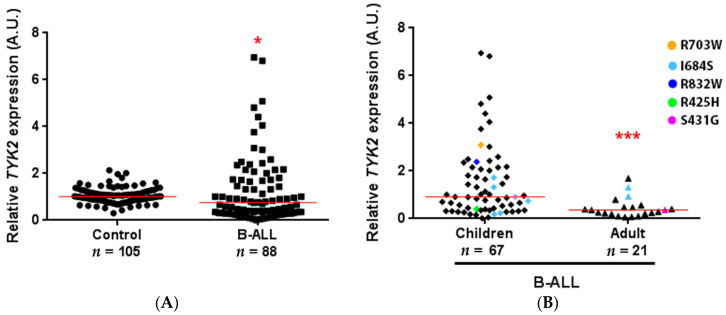
*TYK2* expression in B-cell precursor acute lymphoblastic leukaemia (B-ALL) patients. qPCR was performed on cDNA obtained from 1 μg of RNA isolated from the bone marrow of B-ALL patients and peripheral blood mononuclear cells (PBMCs) of healthy donors. For each sample, data were standardised against *GAPDH* expression. (**A**) Total B-ALL patients compared to healthy donors. Red line shows the median in each group (1.009 vs. 0.7594). (**B**) Age-related *TYK2* expression in B-ALL patients. Children under 18 years compared to adults (median: 0.9147 vs. 0.3609). Patients with TYK2 variants are highlighted with different colours. * *p* < 0.05, *** *p* ≤ 0.001.

TYK2 function could also be affected by changes at the expression level. To test whether *TYK2* expression was altered in B-ALL patients, we performed RT-qPCR in bone marrow samples of 88 B-ALL patients at diagnosis (67 paediatric, age ranging from 1 to 17 years, median 5, mean 6.9; 21 adults, ranging from 21 to 90, median 48, mean 49.9) compared to PBMCs of 105 healthy donors (seven umbilical cords and 98 adults with age ranging from 19 to 66 years (median 33, mean 35.1)) (Figure 6). *TYK2* expression was significantly lower in patients (*p* ≤ 0.05) compared to that in healthy donors (Figure 6A). Twelve (13.64%) of the B-ALL patients analysed showed *TYK2* variants, while 33 (37.5%) did not present any variant and 44 were not sequenced. Regarding the most frequent TYK2 variant (I648S) present in eight of the patients evaluated, it displayed quite a heterogeneous expression pattern. Surprisingly, the two patients bearing TYK2 R425H or S431G had a very low RNA expression level, whereas those with TYK2 R703W or R832W (variants located in the pseudokinase domain) showed high *TYK2* expression (see coloured dots in Figure 6B). However, these few cases were not enough to establish an association between expression level and variant presence. There were significant differences in *TYK2* expression in B-ALL between children under 18 years and adults (*p* ≤ 0.001) (Figure 6B), while no significant differences were found regarding genetics (WHO subgroups), sex or blast cell count (data not shown). To obtain better insight into the relationship between *TYK2* expression and age, a correlation analysis was performed including controls and patients (Figure S5). The results indicated that *TYK2* expression was associated with age in B-ALL patients (rho (ρ) = −0.46; *p* < 0.001) but not in healthy controls (ρ = −0.16; *p* = 0.140). However, the low number of paediatric control samples included in this study due to their low availability must be noted. Overall survival (OS) analysis (Figure S6) considering both *TYK2* expression and age did not reach statistically significant differences (*p* = 0.822). As expected, in our cohort, adults had a 3.7-fold risk of early death compared to children (95% CI = 1.56–8.77, *p* = 0.003).

## 4. Discussion

B-ALL is a heterogeneous malignancy with a broad spectrum of genetic alterations, although one third of B-ALL patients do not have molecular markers associated with the disease. The improvement of cytogenetic and genomic techniques and integration with NGS has enabled detailed characterisation of the genomic complexity of B-ALL. In previous studies it has been reported that a set of mutated gene exons are hot-spot regions for leukaemia (*TP53*, *JAK2*, *PAX5*, *EBF1*, *LEF1*, *IL7Ra*, and *IKFZ*) [4,45]. Within the JAK family, JAK2 gain-of-function mutations such as R683G/S have been associated with B-ALL [4,46]. Although the *TYK2* gene is very polymorphic and more than 100 variants have been described in humans, *TYK2* germline mutations have been recently described in two patients with a secondary B-ALL [19]. These *TYK2* mutations (P760L and G761V) are located in two adjacent codons of the pseudokinase domain and have been proposed, based on in silico predictions, as activating mutations predisposing to the development of ALL. We did not find these mutations in our patient cohort. However, the inactivating cancer-associated germline SNP P1104A, described in independent tumour tissues and in AML [20,21,36], was found in two cases (3.22%). Recently, homozygous P1104A has been described as a common monogenic aetiology factor of tuberculosis [35]. The susceptibility to infection is a hallmark of leukaemia [47], and it is associated with TYK2 deficiency in humans and mice [11,13,14,15]. TYK2 R703W detected in one of our patients has also been found in AML, and neither the impairment of TYK2 phosphorylation nor its effect on TYK2 protein expression was detected when transduced in TYK2-deficient cells [21]. In addition, the missense TYK2 I684S, described as producing a catalytically impaired kinase [25] and associated to protection against autoimmune diseases [48], was detected in eleven patients (17.74%) of our sequenced cohort, matching the frequency reported for AML patients [21], which is threefold the frequency found in T-ALL samples [32]. There are some discrepancies related to the functional impact of the I684S substitution. While TYK2 deficient cells overexpressing this variant show catalytically impaired protein [21,25], HEK293 cells modified by CRISP-Cas9 to express it show no differences in TYK2 or STAT phosphorylation or in the expression of TYK2 protein with respect to parental cells [34].

The VAF for *TYK2* variants detected in our patients suggests that they are heterozygous in blast cells. Unfortunately, our study was retrospective and other tissues were no longer available to determine whether they are also present in non-tumour cells. Interestingly, TYK2 R425H was previously found in the T-ALL cell line MOLT-16, but not in the T-ALL patients analysed, and was reported to be unable to transform murine pro-B Ba/F3 cells to IL-3 independent growth [32]. In this study, its capability to be phosphorylated and to activate STAT1/2, but not STAT3, and mediate target gene induction in response to IFNα, is proven. Furthermore, even though the TYK2 R832W clone presented lower expression than the WT, the protein was able to be phosphorylated and induce earlier STAT target gene expression (1 h) when stimulated by IFNα, similarly to the hyperactive V678F. Finally, TYK2 S431G is also functionally impaired in vitro, and cells carrying this mutation poorly induce STAT-target gene expression in response to IFNα stimulation, similarly to what is observed for the kinase-negative protein TYK2 K930R. Despite having the ATP binding site mutated, TYK2 K930R is still phosphorylated upon treatment with high doses of IFNα and allows some inducible gene expression due to JAK1-mediated cross-phosphorylation of conserved tyrosine residues within the activation loop of the kinase domain [25,49].

From a structural point of view, TYK2 R425H and S431G are located in the JH4 region within the FERM domain, which regulates the catalytic activity of the protein and has been implicated in the interaction with cytokine receptors [6]. It has been described that the 591 amino-terminal residues of TYK2 maintain the steady-state level of IFNAR1 protein in the cell [43] with the main interaction surface within JH7-6 regions. Additionally, the JH5-4-3 domains contribute to the in vivo assembly of TYK2 and IFNAR1 [50]. However, TYK2 residues 365–497 (JH4 region) transiently transfected in 293T cells did not show binding to the IFNAR1 cytoplasmic domain [51]. Accordingly, our dynamic structural analysis of TYK2–IFNAR1 interaction suggests that the mutation in residue 431 does not affect the interaction with IFNAR1. Interestingly, these TYK2 variants in the FERM and pseudokinase domains similarly impair kinase activity, suggesting that signalling through the JAK–STAT pathway is regulated on multiple levels, including intramolecular and intermolecular regulation [48].

Finally, it has been reported that TYK2 deficiency in mice increases the susceptibility to develop Abelson-induced B lymphoid leukaemia/lymphoma linked to a diminished cytotoxic capacity of Natural Killer (NK) and NKT cells as a consequence of decreased IFN-γ production [16]. In addition, TYK2 is crucial for cytotoxic T-lymphocyte-mediated immune surveillance [52], therefore the low expression showed in B-ALL samples might unbalance this function. On the other hand, while TYK2 kinase activity is required for functional type I interferon responses in vivo [53], expression of the kinase-inactive mutant TYK2 K923E partially rescues NK cell-mediated anti-tumour response, thus supporting kinase-independent functions of TYK2 [54] that we cannot rule out for the variants analysed in the present study. At the same time, TYK2 plays an important role in B-cell development by mediating IFNα/β-induced apoptosis of primary pro-B cells, which does not occur in TYK2-deficient mice [15,55]. To the best of our knowledge, this is the first analysis of *TYK2* expression levels in immature human ALL-blasts, which is lower than in healthy PBMC. Further refinements to ascertain whether *TYK2* expression is reduced in ALL cells as compared to that in normal precursor-B-cells would require samples not available at this point, and therefore this question remains to be elucidated. On the other hand, the described level of *TYK2* expression in total PBMC (https://www.proteinatlas.org/ENSG00000105397-TYK2/blood) is lower compared to that in granulocytes, monocytes, or dendritic cells; similar to that in lymphocytes; and slightly higher than those of B-cell plasmablasts [56]. Further investigation should be performed to assess whether the low *TYK2* expression found in ALL-blasts could favour the survival of leukaemic blasts in response to apoptotic signals and to define the role of these changes in B cell leukaemic disease.

## 5. Conclusions

In this study, we characterised a novel *TYK2* variant (S431G), together with two rare polymorphisms (R425H and R832W) found in B-ALL patients. These TYK2 variants presented differences in IFN signalling response and in STAT-target genes induction and none of them phosphorylated STAT3 in in vitro kinase assays. Molecular dynamic analysis of TYK2 variants located in the FERM domain (R425H and S431G) showed that the interaction with IFNAR1 was not affected by these point-mutations. Finally, *TYK2* expression was lower in B-ALL patients compared to healthy controls. In summary, we provide evidence of naturally occurring catalytic loss-of-function TYK2 variants and reduced expression of TYK2 in B-ALL patients and propose that both mechanisms could contribute to facilitate tumour expansion.

## Figures and Tables

**Table 1:** Disease-associated non-synonymous TYK2 variants.

rs	Ancestral > Derived ^a^	Location (Chr)	EUR MAF ^b^	Protein Change (Domain)	Number of Patients	Disease Association	Ref.
150601734	G > A	19:10364707	0.000008 (A) ^c^	p.R425H (FERM)	1	T-ALL cell line	[32]
-	A > G	19:10364724	--	p.S431G (FERM)	1	--	
12720356	A > C	19:10359299	0.092 (C)	p.I684S (pseudokinase)	11	Protects against RA and autoimmunity; AML, T-ALL cell line	[21,32,33]
55882956	G > A	19:10359243	0.001 (A)	p.R703W (pseudokinase)	1	AML	[21]
144995884	G > A	19:10356691	0.0 (A)	p.R832W (pseudokinase)	1	--	
34536443	G > C	19:10352442	0.029 (C)	p.P1104A (kinase)	2 ^d^	Decreased susceptibility to RA and autoimmunity Susceptibility to mycobacteria NF1-PNSTs and tumour tissues	[20,34,35,36,37,38]
55886939	T > C	19:10350910	0.004 (C)	p.E1163G (kinase)	1	T-ALL cell line	[32]

^a^ Refers to the (-) strand nucleotide. ^b^ Minor allele frequency in Europeans (1000 Genomes), according to Ensembl database (https://www.ensembl.org/). ^c^ Minor allele frequency (ExAC). ^d^ Two patients who also bear the pI684S variant. T-ALL: T-cell acute lymphoblastic leukaemia; AML: acute myeloid leukaemia; RA: rheumatoid arthritis; NF1-PNSTs: neurofibromatosis type 1-associated malignant peripheral nerve sheath tumours.

## Supplementary Materials

The following are available online at https://www.mdpi.com/2073-4425/11/12/1434/s1, Table S1: Sequences of primers used to amplify *TYK2* in the NGS study, Table S2: Clinical data of patients with TYK2 non-synonymous variants, Table S3: Calculation of the theoretical energy components from the trajectories of molecular dynamics, Figure S1: Auto-phosphorylation activity of TYK2 variants and comparison of TYK2 expression in selected cell clones, Figure S2: Relative quantification of three replicates of TYK2 variant functional analysis, Figure S3: Molecular modelling of the superposition of FERM, SH2, and pseudokinase domains on TYK2 protein, Figure S4: Total energy time course of TYK2 WT–IFNAR1, TYK2 R425H–IFNAR, and TYK2 S431G–IFNAR1 complexes, Figure S5: Association between *TYK2* expression and age, Figure S6: Overall survival of 88 B-ALL patients.
