# Supplementary material for: TYK2 Variants in B-Acute Lymphoblastic Leukaemia
Source: Genes (Basel). 2020 Nov 28;11(12):1434. doi: 10.3390/genes11121434 (PMC7761059; doi:10.3390/genes11121434)
Supplement: Supplementary file 1 [file genes-11-01434-s001.pdf]

Table S1. Sequences of primers used to amplify *TYK2* in the NGS study

Amplicon ID	Ion AmpliSeq Forward Primer	Ion AmpliSeq Reverse Primer	Amplicon Start[a]	Amplicon Stop
7153692681	CCTCAAGATCATGGTTAGGC TCA	CCTCATACCCATTCTGAAGACAGTC	10461312	10461582
7153692680	GGAGACCAGACTCCCAGCTAA	CACCTCTGTGAACTTATTTTTCTTTC TTGG	10461145	10461368
7153692679	GTGCTCCCATCCAAGTGCA	AATGGGCGTGTGCGTTTC	10491084	10491302
7153692678	CAGGGTCGGAGTGAAGTTTG	CGGAGGTCCTCAGGAAGAAG	10490948	10491151
7153692676	GGTTGTAATGTTTCAATATAG TTCCGCAT	AGGAGAATTGTGGCCTGAAAGAG	10490405	10490560
7153692675	GTAAAATAAAAATCTGCTCT TAACACACCTCC	CAGGGTCTGTGCTGAATGTGTAAT	10490221	10490458
7153692672	CCCAGACTCACCAACTTTATG TG	GGTCTCTGGGCTGAGACTTG	10488878	10489149
7153193306	GCTCGAGTGTGGCTAGGTA	GTTAAGCCACTTGCCCTGTG	10476410	10476654
7153193297	CTCCTTGTTCACCTCCTCCTCTA	CCAGCAGATGGTCATGGTCAAA	10476195	10476451
7153193290	CCTCAGAGGCTAGGGTCAAG	AGACCCTGGCCCTGAGTCTG	10476107	10476301
7153193266	GGGCGATGTCATGGTGACTAG	AGATCAGCTGCTAACTTTCACCATG	10472447	10472685
7153193238	AGAGTCTCTAATTGGCTAGGC CA	GCTCTACCACGAGCACATCATC	10464614	10464767
7153193175	CAAAGGTCTCCACCCACCTTA	ACGGTAGCAAATGACGTGACT	10478953	10479142
7153193142	GCTCTCATTCTTAAAGTGGTG GATCT	GATACCTCTGGGCTAGAGAGGAA	10477172	10477322
7153193133	AAAAAGTAGAGGCACGGCAAT ATG	GTTTGTGAATGACGTGGCATCA	10476991	10477245
7153193109	CGTCAAAGCAGATCTCCAGG AG	TCTCCAGGGAGGGTGAGTAC	10468474	10468690
7153193103	TGGTAGCCCAGAGAGACTTG AA	CTAAGCACCGCCATGGACAAGT	10468313	10468535
7153193037	CATGATGATGAGATTGGAGGT TTCTG	CCAGCGTTCGGGAACTTG	10472834	10473038
7153193033	ACAGAATACCGCCATGGTG AA	CCCTCCATGACTTGATGCCT	10472648	10472885
7153193012	CCTGCTCATACCTGCTCAAA GA	GCTGGTCTGACTCTGTGCTAAG	10478719	10478927
7153104521	AGACCTGGCTCATGAGGCT	CCCTAGTCACCATGACATCGC	10472237	10472470
7153104502	GGTCTAGGTTGAAGGTCAAGGT	CACAGGCCTTCTACGAGACA	10472020	10472279
7153104476	CCACACACCAGGTAGCTGAG	AAGTCCCTCCCTGGCGTCT	10469842	10470065
7153104453	AGGGCGAAACTCCACCTAAAAC	CCCATGGCTTGGAAGATGGT	10469720	10469907
7153104383	CATGCTTATGAATGCCACTGCAA	CAGTGTCTGACCTATGAGCCAA	10467103	10467322
7153104356	CTACTCCACCCTGCCTGTTC	CCCACTGAAACTCACGAGC	10463537	10463809
7153104324	GGCCACACACATTACCATGAAC	CCAGTGATAGTCACAGTTGTCCTT TTC	10468777	10468928
7153104297	CAGAGGTCACCAAGGGTGAAAG	CACCTCAGGAGAACAAGAACCTG	10468609	10468822
7153104240	GTGCTCCAGTGAATGAGGTACAG	CCTCTAAGTAGGTAATGAGTGTC ATTGTT	10473270	10473423
7153104219	CGTCCTGCTGCTCAATGGG	ATTTGTGCAGGCCAAGCTG	10473074	10473327
7153104090	CGCCCAGATCTCGGATCTTTTT	AGTTTTCTTACCTCTAGAATCGGAT CCT	10465189	10465404
7153104057	CCAGGCAGATCCTTTCGGAATA	CGGTTTTCCACAAGCGCTATTTG	10464992	10465234
7153103962	CCTGGCCTTGGTACTTCTCATG	GAGACTTCCTTTGTCTTTCCCTGA	10461535	10461725

Amplicon ID	Ion AmpliSeq Forward Primer	Ion AmpliSeq Reverse Primer	Amplicon Start[a]	Amplicon Stop
7153103724	GTTGGTCGGATCGTAGCAGTAC	CCAAGGAGTCTTAATAGAGCGGAGTA	10464865	10465054
7153103694	CGCAGCAGCCCTTGTACTT	ACTTCGGCAAGGTCAGCTT	10464726	10464906
7153103626	CCCAGCCTATGCCTTTCTAATTG	GGGTCCTTCAGTCTCAGGTGA	10463045	10463320
7153103564	CCATCCCGGATGCTCATCAC	TGAAAGAGCACTGTGTCAGCAT	10475307	10475573
7153103445	GGCCGTCAGGCGGAAATA	CTGTGACTTCCGGGACATCA	10475372	10475605
7153103408	GTCTCCCAGCAGTTCTTCATGA	CCTGCCTTTCATTGCCTCTTGA	10461612	10461887
7153103317	CGACCAACCTCGCAGATCT	ACCTGAGAACTGGGTCTAGTGT	10464195	10464458
7153103290	GTGTGGCCAAGCAAGCCAAAC	CAGCTGGTCATGGAGTACGT	10464038	10464308
7153094479	GCTGCCTCTGGTAGAAATGCTC	CCTCATCTGTATAATGGAACTGATAAGAGC	10467369	10467525
7153094476	CGCAGGATGGTGCGGAAT	TGGGTCCCTTTCCCAACAGA	10467269	10467413
7153094389	CCCTAGGGCTCACAGTCTAGTT	CGACTCCAGCCACTACCTGT	10475222	10475373
7153094366	CCCAGATAGCATGAGTTGAAACCT	GACCCAGCCTCATTTGAGTACC	10478613	10478763

[a]Human Feb. 2009 (GRCh37/hg19) Genome Assembly. Human *TYK2* is located in chromosome 19. The following sequences are not covered by these amplicons: in exon 11 from G530 to P506; in exon 8 from T384 to G338; in exon 3 from V64 to A61.

Table S2. Clinical data of patients with *TYK2* non-synonymous variants

Patient ID	Age years/Sex	Risk Group	Frontline therapy	EGIL	Cytogenetic subgroup	Bone marrow blasts %	TYK2 variant	VAF %	Clinical status
1	3/F	UNK	SHOP-2005	ALL-B II	TCF3(E2A)-PBX1	90	R425H	48	CCR-A
2	46/F	HR	Idarrubicine-(Ara-C)	MPAL	Hyper (47-50)	50	S431G	36	Rel-D
3	14/F	HR	SHOP-2005	ALL-B II	Normal	97	I684S<br>P1104A	47<br>32	CCR-A
4	7/F	UNK	SHOP-2005	ALL-B	TEL-AML1	UNK	I684S	49	CCR-A
5	9/F	HR	LAL-SHOP-2005 HR	ALL-B III	Low hyperdiploid (47-50)	UNK	I684S	49	CCR-A
6	8/F	IR	PETHEMA LAL-RI/96	ALL-B III	Others	90	I684S	51	CCR-A
7	13/M	HR	SHOP-2005	ALL-B II	Low hyperdiploid (47-50)	92	I684S	49	CCR-A
8	68/F	HR	PETHEMA OLD-07 HR PH+	ALL-B	*BCR-ABL1*	92	I684S	46	Manteinance Imatinib
9	5/F	LR	PETHEMA LAL-BR 2001	ALL-B II	High hyperdiploid (>50)	52	I684S	50	CCR-A
10	93/F	HR	UNK	ALL-B III	*BCR-ABL1*	UNK	I684S<br>P1104A	49<br>51	Rel-D
11	11/M	HR	SHOP-2005	ALL-B II	High hypodiploid (44-45)	95	I684S	54	CCR-A
12	2/M	UNK	SHOP-2005	ALL-B III	Not evaluable	99	I684S	54	UNK
13	52/M	HR	UNK	ALL-B III	High hyperdiploid (>50)	UNK	I684S	48	Rel-D
14	6/F	UNK	SHOP-99	ALL-B III	Normal cytogenetics	95	R730W	49	CCR-A
15	4/F	UNK	SHOP-2005	ALL-B II	Not evaluable	90	R832W	30	CCR-A
16	10/F	HR	SHOP-2005	ALL-B II	TCF3(E2A)-PBX1	100	E1163G	47	Rel-D

VAF: Variant allele frequency; M, male; F, female, LR, low risk; IR, intermediate risk; HR, high risk; UNK, unknown; N, no; CCR, continuous complete remission; R, Relapse; D, dead; A, alive. EGIL, European Group for the Immunological Classification of Leukaemias. Patients were treated according to PETHEMA (Spanish Programme for Haematology Treatments) and SEHOP (Spanish Society of Haematology and Paediatric Oncology) risk-adapted protocols. Risk-group stratification was established according to PETHEMA protocols based on age, white blood cell count and cytogenetic subgroup.

Table S3. Calculation of the theoretical energy components from the trajectories of molecular dynamics.

TYK2	ΔE_{vdw}	ΔE_{ele}	ΔG_{GB}	ΔG_{solv}	ΔG_{bind}
WT	−156.13 ± 12.0	−557.30 ± 78.6	617.64 ± 68.3	-22.05 ± 1.7	−117.85 ± 12.4
R425H	−156.87 ± 12.0	−612.72 ± 78.6	669.18 ± 69.9	-22.39 ± 1.3	−122.81 ± 10.0
S431G	−152.42 ± 9.4	−666.01 ± 65.9	713.94 ± 62.7	-21.99 ± 1.0	−126.49 ± 12.6

ΔE_{vdw}, contributions of van der Waals interactions; ΔE_{ele}, electrostatic energy; ΔG_{GB}, polar solvation energy; ΔG_{solv}, desolvation free energy ($\Delta G_{solv} = \Delta G_{GB} + \Delta G_{nonpol}$); ΔG_{bind}, binding affinity.

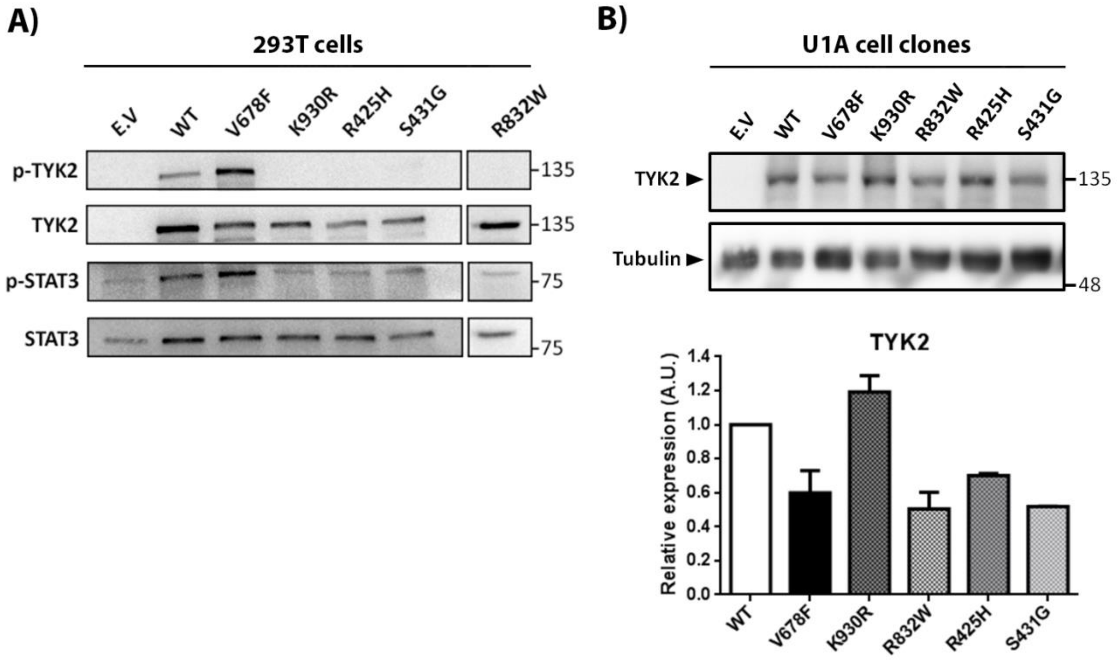

Figure S1. Auto-phosphorylation activity of TYK2 variants and comparison of TYK2 expression in selected cell clones. A) Basal auto-phosphorylation state. 293T cells were transiently transfected with empty vector (EV) or TYK2 variants. Cell lysates were analysed by Western blot with anti pTYK2 Ab specific to phospho-Tyr1054/Tyr1055 in the activation loop, anti pSTAT3 (Y705), anti TYK2 and STAT3 mAb. B) TYK2 expression in selected stably transfected TYK2-deficient UA1 cell clones (upper panel) and relative quantification (lower panel).

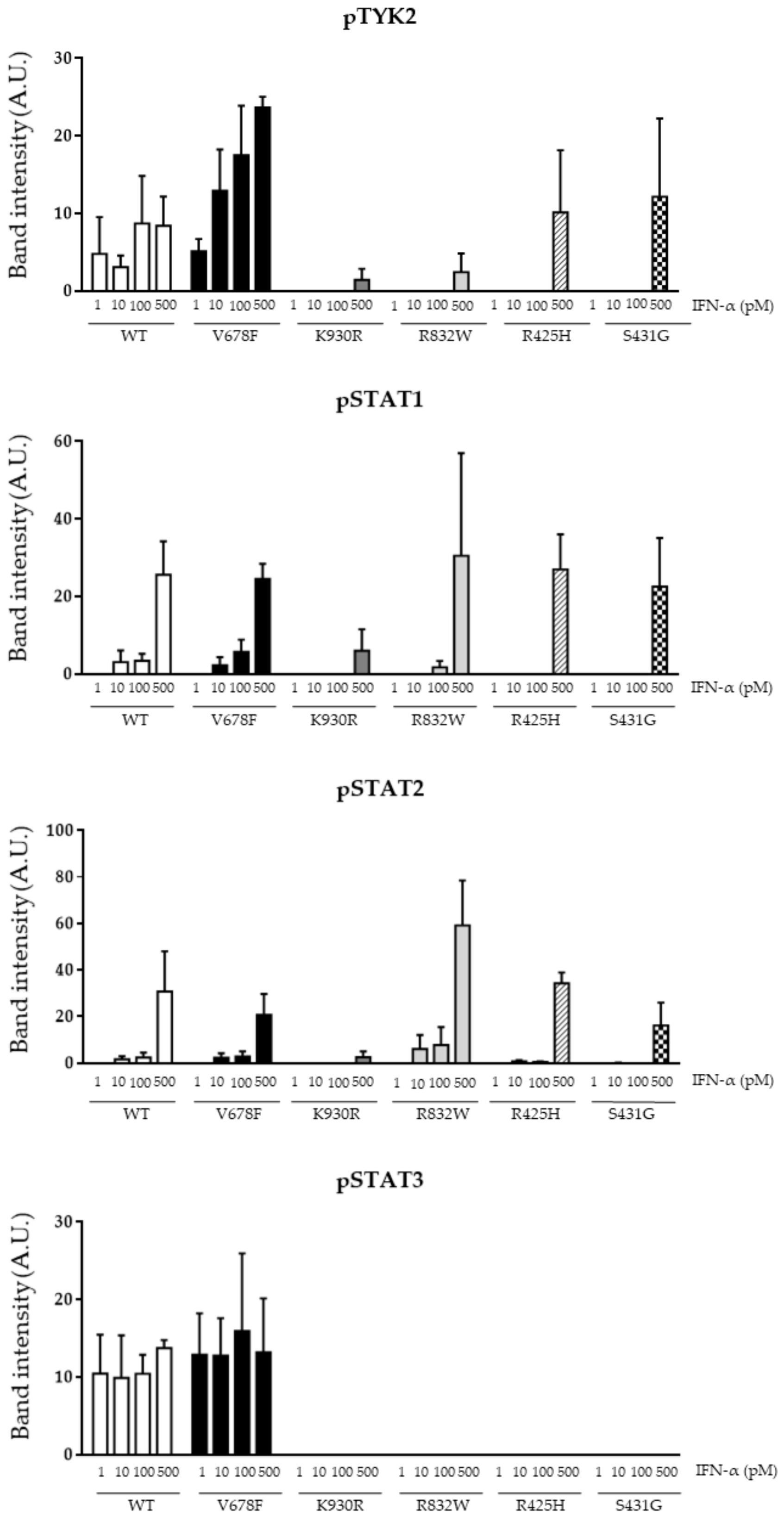

Figure S2. Quantification of pTyk2 and pStat1-3 from western blot analysis of TYK2 variants in response to IFN-α. The level of phosphorylated proteins was normalized to TYK2 total protein and then to tubulin. Mean ± SEM (n=3) is shown in arbitrary units (A.U.).

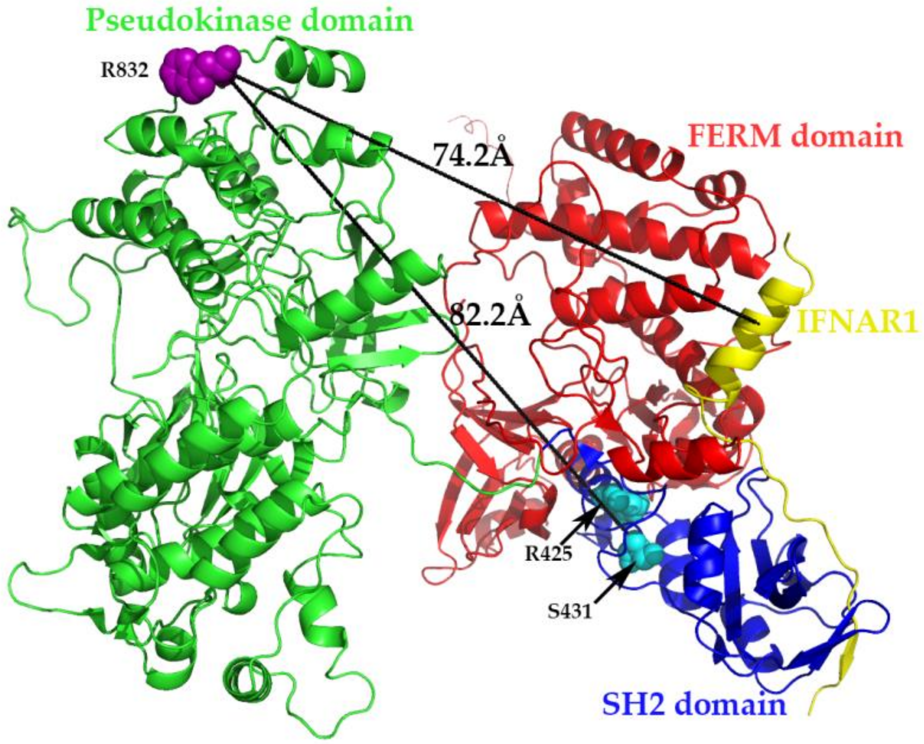

Figure S3. Molecular modelling of the superposition of FERM, SH2 and pseudokinase domains on TYK2 protein. The molecular modelling of TYK2 protein was obtained from Phyre2 (www.sbg.bio.ic.ac.uk)* and TYK2 FERM and SH2 domains with IFNAR1 from the Protein Data Bank (ID PDB: 4PO6). The FERM domain is represented in red, SH2 domain in blue, and IFNAR1 in yellow. Pseudokinase domain (ID PDB: 3ZON) represented in green; the sites of TYK2 R425, S431, and R832 are represented with spheres.

* Kelley LA et al. The Phyre2 web portal for protein modelling, prediction and analysis. Nature Protocols 10, 845-858 (2015)

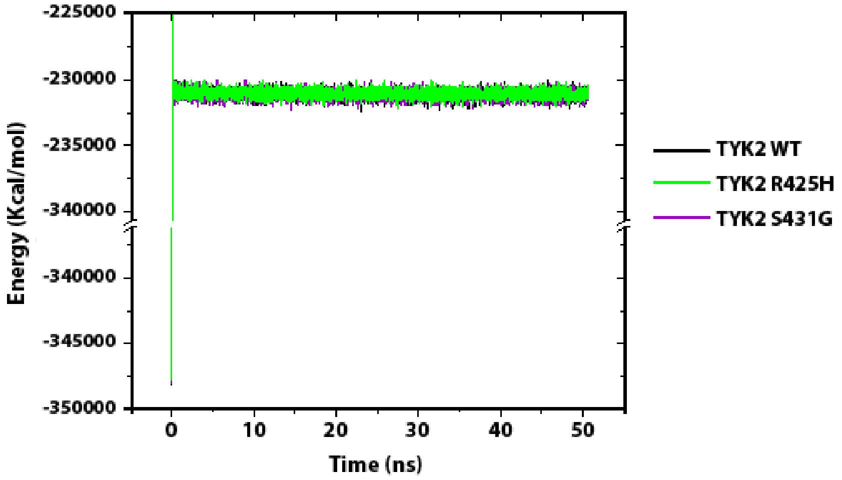

Figure S4. Total energy time course of TYK2 WT-IFNAR1, TYK2 R425H-IFNAR and TYK2 S431G-IFNAR1 complexes. Total energy was monitored as a function of time during the molecular dynamics trajectory, showing that from 10ns it remains stable.

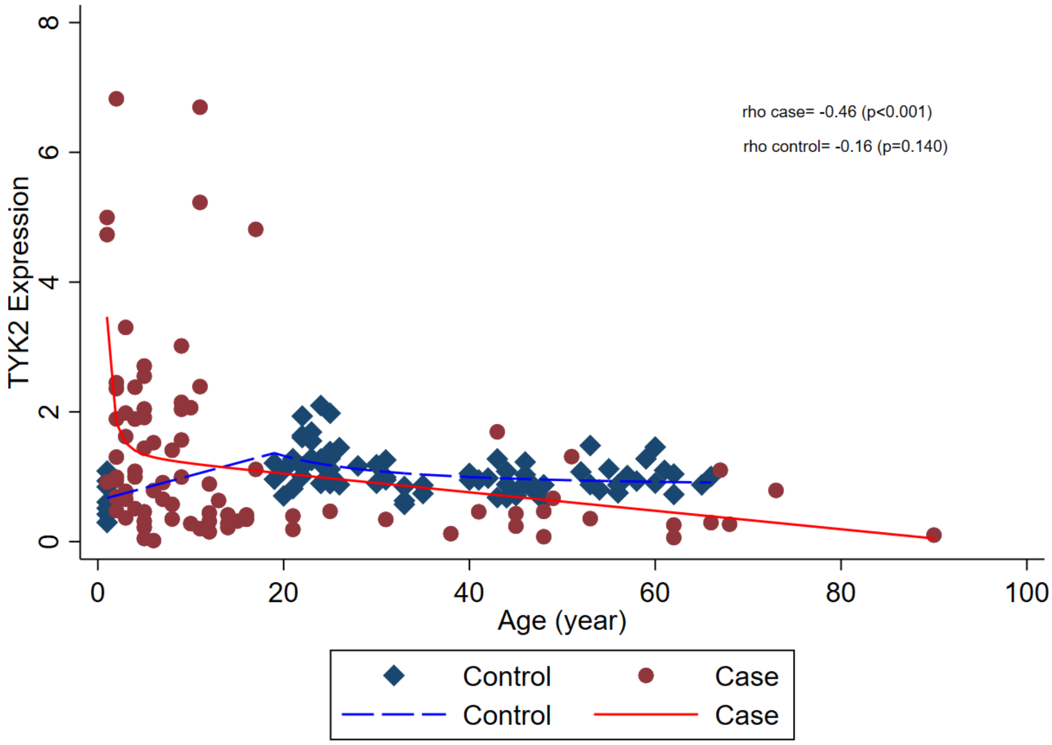

Figure S5. Association between *TYK2* expression and age. Scatter plot represents *TYK2* expression and age of patients (Fit curve in red) and healthy controls (blue). Association was estimated using Spearman correlation. Correlation coefficient (rho) is show inside the graph.

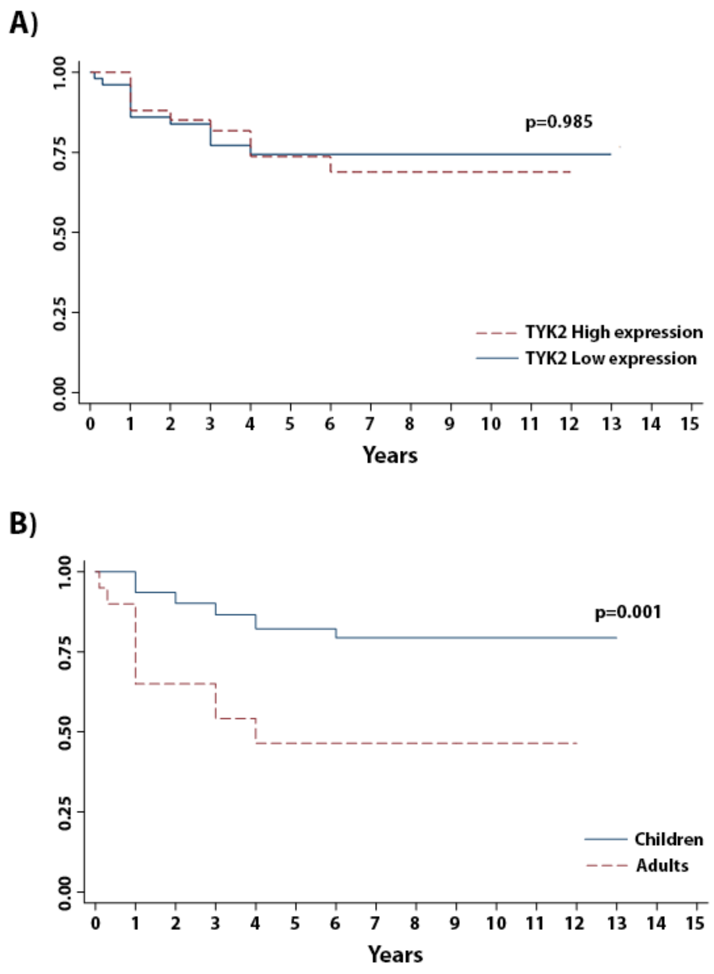

Figure S6. Overall survival of 88 B-ALL patients. A) Overall survival by TYK2 expression. No significant differences were found between the survival time of patients with low (< 1.009) and high *TYK2* expression (> 1.009). This cut-off value is the median obtained in control samples. B) Overall survival by age. Adults presented worse outcome (p≤0.001). Overall survival was represented by Kaplan-Meier curves. Curves were compared by log-rank tests. P values <0.05 were considered statistically significant.
